# Impact of hyponatremia in preeclamptic patients with severe features

**DOI:** 10.1371/journal.pone.0302019

**Published:** 2024-07-08

**Authors:** Kodi M. Endres, Catherine M. Roberts, Xinying Fang, Shouhao Zhou, Tonya S. Wright, Conrad Krawiec

**Affiliations:** 1 Anesthesiology, Penn State Health Milton S. Hershey Medical Center, Hershey, Pennsylvania, United States of America; 2 Obstetrics and Gynecology, University of Pittsburgh Medical Center, Harrisburg, Pennsylvania, United States of America; 3 Public Health Sciences, Division of Biostatistics and Bioinformatics, Pennsylvania State University College of Medicine, Hershey, Pennsylvania, United States of America; 4 Obstetrics and Gynecology, Division of Women’s Health, Penn State Health Milton S. Hershey Medical Center, Hershey, Pennsylvania, United States of America; 5 Pediatrics, Division of Pediatric Critical Care Medicine, Penn State Health Children’s Hospital, Hershey, Pennsylvania, United States of America; Universita Politecnica delle Marche, ITALY

## Abstract

Hyponatremia, though common in women with preeclampsia, has not been well studied. Our primary objectives are to assess the clinical characteristics and emergency therapy applied to subjects diagnosed with preeclampsia. We hypothesize that hyponatremia present in preeclamptic patients with severe features is associated with greater use of emergency hypertensives, antenatal steroids, and cesarean delivery. This is a retrospective descriptive study utilizing an electronic health record database (TriNetX ®). We collected and evaluated the following data of subjects aged 15 to 54 years with preeclampsia with severe features diagnosis: demographics, diagnostic codes, medication codes, procedure codes, deaths, and laboratory results. A total of 2,901 subjects [215 (7.4%)] with a sodium level below 134 mEq/L and [2686 (92.6%)] with a sodium level above 135 mEq/L were included. A higher proportion of subjects in the below 134 sodium group received emergency antihypertensives [165 (76.7%) versus 1811 (67.4%), p = 0.01], antenatal steroids [103 (47.9%) versus 953 (35.5%), p = 0.001], and cesarean section [27 (12.6%) versus 97 (3.6%), p = <0.001]. We found that hyponatremia may be associated with emergency antihypertensive use, antenatal steroid use, and cesarean section in patients with preeclampsia with severe features. Future research is needed to determine if routine sodium levels assessed in preeclamptic subjects with severe features identify subjects at risk of receiving these treatments.

## Introduction

Preeclampsia is estimated to affect up to 8% of pregnancies worldwide and can be life threatening to both mother and fetus [1]. It is among the major causes of maternal death and morbidity in the United States [2–5]. Additionally, because the placenta is thought to be impacted by preeclampsia, fetuses are at risk for intrauterine growth restriction, leading to increased perinatal morbidity and mortality [6].

Preeclampsia is a clinical diagnosis, thus several criteria are utilized to make this determination. The patient must be greater than 20 weeks gestation (or in the postpartum period), and have new onset hypertension with either proteinuria or end organ dysfunction [1, 7]. At times, preeclampsia may manifest with severe features, which requires urgent evaluation and treatment [1]. This includes severely elevated systolic (160 mm Hg and above) or diastolic (110 mm Hg and above) blood pressures, development of neurologic symptoms (i.e. headaches, visual changes), and end organ dysfunction [1]. Because preeclampsia can impact multiple organs, it can be diagnosed clinically and in conjunction with laboratory testing. These tests include a urine protein/creatinine ratio or 24- hour urine collection for proteinuria, a platelet count, renal function, and hepatic function testing [1]. These tests, in combination with physical exam and clinical findings, can assist the clinician in diagnosing preeclampsia, and may also help to determine the severity of disease. By identifying the risk factors associated with developing severe features of preeclampsia early, clinicians can potentially minimize negative maternal and fetal outcomes.

Hyponatremia is known to occur in expectant women. In normal pregnancy, plasma osmolality decreases, resulting in a physiologic reduction in plasma sodium concentration [8, 9]. The sodium levels may fall as low as 130 mEq/L and there may be no adverse consequences [8, 9]. When serum sodium concentrations decrease below 130 mEq/L, however, it can be considered pathologic and the patient may be at risk for complications related to preeclampsia [9, 10]. It is unknown, however, if any type of hyponatremia is clinically significant, particularly in acute obstetrical situations. An understanding of how the presence of hyponatremia impacts patients clinically can possibly assist in improving the approach to preeclamptic patients with severe features.

The objective of this study is to utilize an electronic health record (EHR) longitudinal database in order to evaluate the (1) the clinical characteristics, (2) the frequency of emergency antihypertensive and antenatal steroid use, (3) need for cesarean section, and (4) correlation with other laboratory (hepatic function, complete blood count, and renal function) and vital sign (systolic blood pressure) abnormalities of subjects diagnosed with preeclampsia with severe features and hyponatremia. We hypothesized that when hyponatremia is present in preeclamptic subjects with severe features, it is associated with greater use of emergency hypertensives, antenatal steroids, need for cesarean delivery, and is correlated with other laboratory and vital sign abnormalities.

## Materials and methods

### Study design

This is a retrospective observational cohort study completed using the TriNetX® (Cambridge, Massachusetts, USA, https://trinetx.com/) EHR data of pregnant women with a first-time preeclampsia with severe features related diagnostic code.

### Data source

TriNetX® is a global multi-center longitudinal database, based largely in the United States, that utilizes proprietary data processing methodology to collect, organize, and put forth frequently updated EHR data to researchers in a user-friendly fashion. It includes demographic characteristics as well as diagnostic, procedure, and medication codes, and laboratory values (when available). TriNetX ® database details have been previously described in the literature, including how it protects healthcare data and its strategy for maintaining compliance with the Health Insurance Portability and Accountability Act (HIPAA) security and privacy rules [11, 12].

### Ethics statement

Since no protected health information is obtained by the user, the Penn State Health Institutional Review Board provided a waiver of informed consent and deemed this study non-human subject research.

### Inclusion and exclusion criteria

On July 25^th^, 2021, we analyzed available EHR data of 2,901 pregnant females age 15 to 54 who had a preeclampsia with severe features related International Classification of Diseases 10^th^ edition (ICD 10) diagnostic code and were coded to have obtained the following services: (1) magnesium therapy and (2) sodium level laboratory evaluation. Because magnesium is administered for seizure prophylaxis in preeclamptic patients with severe features, this assisted in ensuring we only included this patient population [1]. The index date of this study was the date of when the subject met all the inclusion criteria and the timeframe was September 30^th^, 2015 until May 19^th^, 2021

### Data collection

After the query, we evaluated the following data: age, race, ethnicity, body mass index, systolic blood pressure, sodium, and preeclampsia specific laboratory codes/results [alanine transaminase (ALT), aspartate aminotransferase (AST), creatinine, hematocrit, platelet count), medication codes (emergency anti-hypertensives, aspirin presence, vasoactive use, antenatal steroids), procedure codes (need for critical care services, mechanical ventilation, cesarean section), and deaths. [Please see S1 Table for diagnostic, procedural, medication, and laboratory codes and definitions].

### Variables

Patient characteristics (age, sex, race, ethnicity, body mass index) were reported. Deaths were reported if they occurred within 30 days of the index date. The primary outcomes of interest were the need for use of emergency anti-hypertensives, aspirin use, vasoactive use, antenatal steroid use, critical care services, and mechanical ventilation 7 days after the index date (to ensure all that all preeclampsia associated interventions were captured). Sodium, and preeclampsia specific laboratory codes/results [alanine transaminase (ALT), aspartate aminotransferase (AST), creatinine, hematocrit, platelet count) were analyzed on the index date.

Exact death dates were not for patient privacy reasons (only the month and year). In order to define death within 30 days, we first added 30 days to the index date. If the month and year of the death date provided was equal to this calculation, then the patient met the definition of death. For example, if the patient’s index date was January 1, 2020, the patient would have been classified as a death within 30 days if the death date was reported as January 2020, but not, if the date was reported as February 2020. Similarly, exact birth dates were not provided, only the year of birth. Thus, ages that were reported for this study are approximate. For example, a subject born in 1996 with an index date of January 1^st^, 2020, the subject was calculated to be 24 years of age.

Timing of laboratory results are not provided by the TriNetX® database, thus the minimum sodium, hematocrit, and platelet level results and maximum ALT, AST, creatinine were selected for each subject.

We determined the proportion of subjects whose results were within normal limits versus those that were not within normal limits for preeclampsia specific laboratory codes (ALT, AST, creatinine, hematocrit, platelet count), body mass index, and systolic blood pressure for each group.

### Cohort definition

Based on previous studies, the study population was divided into two groups, a minimum sodium level equal to and below 134 mEq/L and a minimum sodium level equal to and above 135 mEq/L [13]. Severe hyponatremia was evaluated and defined as a level below 120 mEq/L.

### Statistical analysis

Summary statistics using mean and standard deviation or proportions were reported for clinical and demographic characteristics of the patients. Chi squared test and Fisher’s exact test were applied to compare race, ethnicity, marital status, death, medications, and procedures between subjects whose minimum Na level is below 134 mEq/L or above 135 mEq/L. Particularly, Fisher’s exact test was applied if at least 20% of the cells had expected counts less than 5 in the contingency tables. For the analysis of medications and procedures, the Benjamini–Hochberg procedure was applied to correct for multiple comparisons. A p value < 0.05 was considered statistically significant.

For laboratory results, we built a correlation matrix to illustrate the distribution of each measurement and the relationship between them. The correlation is measured by the pairwise Spearman’s rho correlation coefficient. Since the maximum AST level and the maximum body mass index each contains 93.97% and 65.12% missing values, we disregarded these two measurements in the correlation analysis. Statistical software R v4.0.5 with packages readxl v1.3.1 and GGally v2.1.2 were used for the data analysis and the construction of the correlation matrix.

## Results

### Demographic characteristics

A total of 2,901 subjects [215 (7.4%)] with a sodium level below 134 mEq/L and [2686 (92.6%)] with a sodium level above 135 mEq/L were included in this study. Race and ethnicity were similar in both groups. There was 1 death (0.5%) for the below 134 sodium group compared to 4 deaths (0.1%) for the above 135 sodium group. One subject (0.5%) had a sodium level of 118 mEq/L. Demographic characteristics are summarized in Table 1. [Table 1]

**Table 1:** Clinical characteristics of subjects with preeclampsia.

	Minimum Na Level 134 mEq/L and Below	Minimum Na Level 135 mEq/L and Above	p value
**Subjects (n, %)**	215 (7.4%)	2686 (92.6%)	
**Age (years, mean, standard deviation)**	30.3 ± 5.7	29.5 ± 6.2	0.067
**Race (n, %)**			0.578
American Indian or Alaska Native	0 (0.0%)	10 (0.4%)	
Asian	13 (6.0%)	108 (4.0%)	
Black or African American	51 (23.7%)	738 (27.5%)	
Native Hawaiian or Other Pacific Islander	0 (0.0%)	4 (0.1%)	
Unknown	19 (8.8%)	226 (8.4%)	
White	132 (61.4%)	1600 (59.6%)	
**Ethnicity (n, %)**			0.066
Not Hispanic or Latina	149 (69.3%)	1693 (63.0%)	
Unknown	33 (15.3%)	400 (14.9%)	
Hispanic or Latina	33 (15.3%)	593 (22.1%)	
**Marital Status (n, %)**			<0.001
Married	27 (12.6%)	67 (2.5%)	
Single	27 (12.6%)	145 (5.4%)	
Unknown	161 (74.9%)	2474 (92.1%)	
**Deaths (n, %)**	1 (0.5%)	4 (0.1%)	0.32

### Medications and procedures

A higher proportion of subjects in the below 134 sodium group received emergency antihypertensives [165 (76.7%) versus 1811 (67.4%), p = 0.01], antenatal steroids [103 (47.9%) versus 953 (35.5%), p = 0.001], and cesarean section [27 (12.6%) versus 97 (3.6%), p = <0.001]. All other medications and procedures were not significantly different between the two groups. [Table 2] [S2 Table].

**Table 2:** Association of sodium level with additional medications and procedures received in subjects with preeclampsia.

	Minimum Na Level 134 mEq/L and Below (n = 215)	Minimum Na Level 135 mEq/L and Above (n = 2686)	p value
**Medications (n, %)**			
Emergency Anti-Hypertensives	165 (76.7%)	1811 (67.4%)	0.01
Aspirin	12 (5.6%)	139 (5.2%)	0.796
Vasoactives	53 (24.7%)	537 (20.0%)	0.137
Antenatal Steroids	103 (47.9%)	953 (35.5%)	0.001
**Procedures (n, %)**			
Critical Care Services	2 (0.9%)	16 (0.6%)	0.39
Mechanical Ventilation	2 (0.9%)	6 (0.2%)	0.171
Cesarean Section	27 (12.6%)	97 (3.6%)	<0.001

### Laboratory results

**Fig 1:**
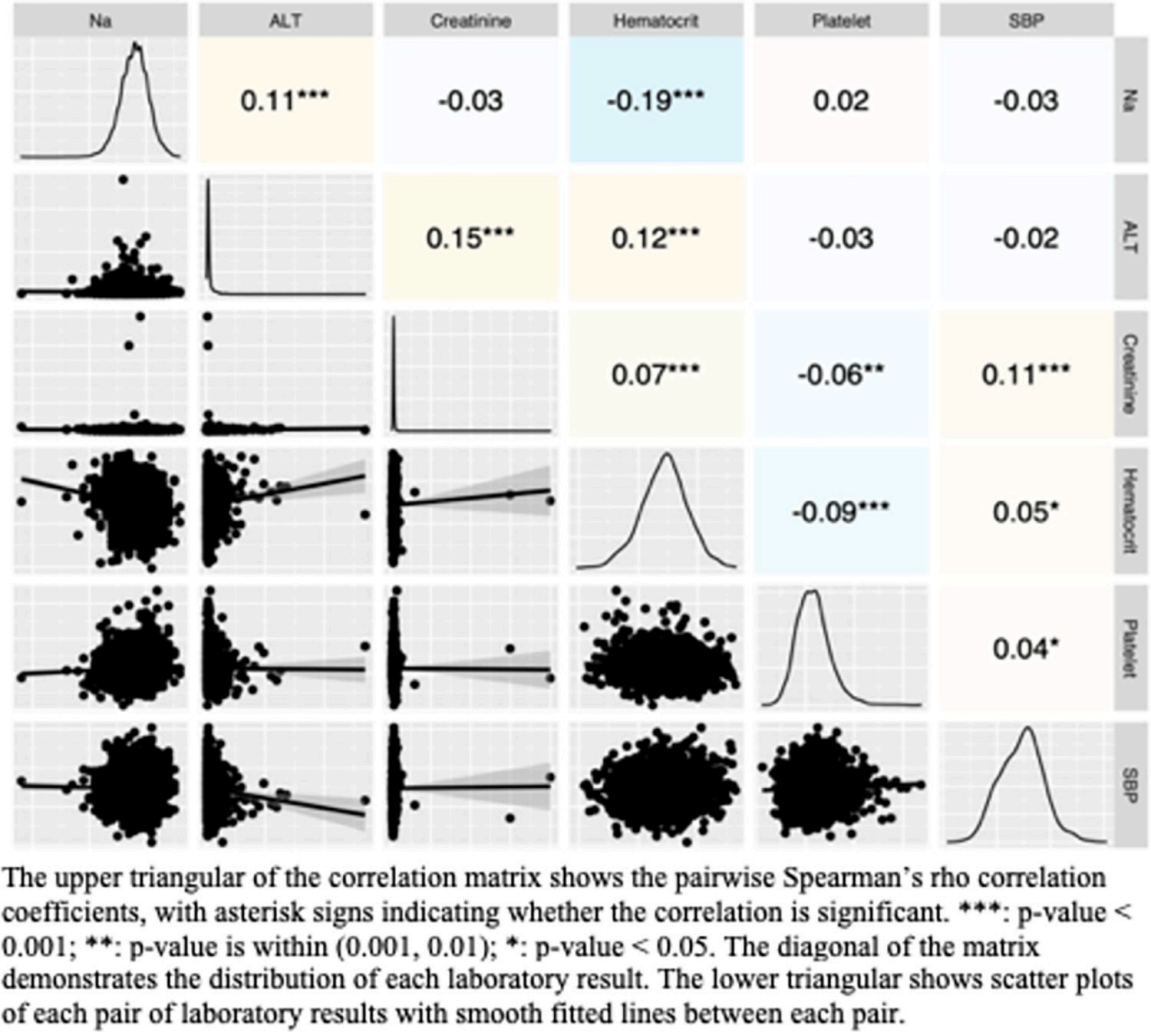
Correlation matrix of subjects with preeclampsia and their laboratory findings.

The following laboratory results were performed for the below 134 sodium group and above 135 sodium group respectively: minimum sodium level (133 ± 1.7) and (138 ± 2.0), maximum ALT level (32.0 ± 42.3) and (28.7 ± 47.8), maximum AST level (25.3 ± 18.6) and (22.7 ± 6.4), maximum creatinine level (0.7 ± 0.3) and (0.7 ± 1.0), maximum hematocrit level (35 ± 9.7) and (34 ± 9.4), maximum platelet count (230.9 ± 90.6) and (232.5 ± 88.1), maximum body mass index (36.1 ± 18.8) and (35.5 ± 17.4), maximum systolic blood pressure (174.9 ± 70.6) and (172.9 ± 57.9). A significant positive correlation was noted between sodium and ALT and a significant negative correlation with hematocrit. [Table 3] [Fig 1]

**Table 3:** Association of mean sodium level with other mean laboratory results in subjects with preeclampsia.

	Minimum Na Level 134 mEq/L and Below (n = 215)	Minimum Na Level 135 mEq/L and Above (n = 2686)
Minimum Na Level	133 ± 1.7	138.4 ± 2.0
Maximum ALT Level	32.0 ± 42.3	28.7 ± 47.8
Maximum AST Level	25.3 ± 18.6	22.7 ± 6.4
Maximum Creatinine Level	0.7 ± 0.3	0.7 ± 1.0
Maximum Hematocrit Level	35.9 ± 9.7	34.3 ± 9.4
Maximum Platelet Count	230.9 ± 90.6	232.5 ± 88.1
Maximum Body Mass Index	36.1 ± 18.8	35.5 ± 17.4
Maximum Systolic Blood Pressure	174.9 ± 70.6	172.9 ± 57.9

## Discussion

In this study, we aimed to investigate the clinical characteristics and association of hyponatremia on the use of emergency antihypertensive therapy, abnormal laboratory values, and vital sign abnormalities in preeclamptic subjects who had severe features. Among a large cohort of preeclamptic subjects with severe features, we found that severe hyponatremia was rare and when hyponatremia occurred (with a cutoff level that is considered physiologic), there was increased need of emergency antihypertensives, antenatal steroids, and cesarean section. Additionally, abnormal lab value associations included a positive correlation between sodium and ALT levels and a negative correlation between sodium and serum hematocrit. There was, however, no increase in mortality and need for critical care services. These findings may have important implications in the assessment and work up of preeclamptic patients who present with severe features.

During pregnancy, there are many physiologic and electrolyte changes that occur. An understanding of these changes, thus, is necessary in order to differentiate it from a pathological disease process. Sodium levels as low as 130 mEq/L may be observed during pregnancy due to a phenomenon known as the “reset osmostat” phenomenon, where an increase in beta human chorionic gonadotropin triggers release of antidiuretic hormone (ADH) sooner than typically [13]. Despite this, treatment is not required because volume status often remains stable [13].

Unlike physiologic hyponatremia in normal pregnancy, however, the etiology and mechanism of severe hyponatremia is not clear. It is theorized that this effect may be a result of syndrome of inappropriate antidiuretic hormone (SIADH) or possibly a compounding effect of preeclampsia and induced nephrotic syndrome [13]. After exclusion of other potential causes of hyponatremia, SIADH can be identified by low serum osmolality and inappropriately elevated urine osmolality and urine sodium concentration [14]. It may be induced by pain, stress or the release of oxytocin in patients with preeclampsia [15, 16]. Another mechanism of SIADH in these patients proposed by Sutton et al is that patients with preeclampsia may have placental abnormalities that impact the production of the placental enzyme vasopressinase that normally would inactivate ADH [17, 18]. In patients with nephrotic syndrome, it is theorized that hyponatremia occurs due to abnormalities in free water regulation resulting in ADH secretion [15, 19]. Even though there are several theories that attempt to explain lower sodium levels, it is unknown if these lower sodium levels are clinically significant in preeclamptic patients.

In our study, we found that hyponatremia was associated with increased use of emergency antihypertensives. The reasons for this are unclear. Hyponatremia is often classified as hypovolemic, euvolemic, or hypervolemic. Because fluid status is not reported within this database, it is possible the patient was hypervolemic resulting in a higher circulating volume, higher blood pressure, and need for blood pressure control [20]. Our study also found that there was a positive correlation between a lower sodium level and ALT. These findings, along with the need for emergency hypertensives, may indicate that a lower sodium level is associated with disease progression in severely preeclamptic patients prompting emergency blood pressure control to avoid cardiovascular complications.

We also found that antenatal steroid use and cesarean section was higher in hyponatremic patients with preeclampsia with severe features. In addition, we found no difference in mortality and need for critical care services between the two different cohorts. As mentioned above, a lower sodium level may be associated with disease progression, thus prompting delivery via cesarean section ensuring maternal well-being. Studies have found that maternal hyponatremia resolved within 48 hours after delivery and it was the severity of the hyponatremia and associated symptoms that necessitated delivery in a majority of these patients [10]. Thus, it may be possible that it was the sodium level itself (and not a clinical progression related to preeclampsia) that resulted in these findings. Regardless of the underlying reason, our findings reinforce the notion that early recognition and intervention may be necessary in order to ensure the best possible outcome for this patient population [15, 21].

Currently, there are no guidelines addressing the assessment and management of hyponatremia in patients with preeclampsia with severe features. Because hyponatremia may be associated with disease progression in severely preeclamptic subjects, routine sodium evaluation should be considered in all patients suspected of having preeclampsia with severe features in conjunction with a thorough and comprehensive history and physical examination [21, 22]. While further research is needed, evaluating a sodium level may not only prompt early recognition, it could also allow for early treatment helping ensure maternal and neonatal well-being (even when a lower sodium level may be considered physiologic in other non-preeclamptic clinical settings)

### Limitations

This study had several limitations. This was a retrospective study, thus the associations we found are not causation. Due to database limitations, clinical documentation and other granular data was not available for review. Thus, we do not know how the patient presented, the risk associated with the patient’s condition, maternal parity, gestational age, as well as the patient’s fluid status. The data was restricted to institutions that participate in this database retrieval system, thus we are limited in the patient data utilized. Furthermore, it is possible that not all EHR data were reported or all subjects who presented with preeclampsia with severe features were coded. Additionally, it is known that maternal hyponatremia affects the neonate as well. Due to database privacy concerns, the TriNetX database does not currently link maternal electronic health data to the neonate. Thus, we are unable to comment on this association and risk of fetal outcomes related to maternal hyponatremia. A cutoff of 134 mEq/L was utilized for the hyponatremia cohort to understand if this value, while considered physiologic, could be potentially a sign of severe illness in preeclamptic subjects with severe features. Future studies may need to utilize lower cutoff levels.

We found that hyponatremia may be associated with a need for emergency antihypertensives, antenatal steroids, and for cesarean section in patients with preeclampsia with severe features. Future research from multiple high-volume centers is needed to determine if routine sodium levels assessed in preeclamptic subjects with severe features identifies subjects at risk of receiving these treatments and to determine the association of specific sodium levels is associated with outcomes.

## Supporting information

S1 Table Diagnostic, procedural, medication, and laboratory codes used to identify clinical characteristics of subjects with preeclampsia. (DOCX)

S2 Table Interventions applied in subjects with preeclampsia divided by year of preeclampsia diagnosis. (DOCX)
