# Supplementary material for: Impact of hyponatremia in preeclamptic patients with severe features
Source: PLoS One. 2024 Jul 8;19(7):e0302019. doi: 10.1371/journal.pone.0302019 (PMC11230559; doi:10.1371/journal.pone.0302019)
Supplement: S1 Table — (DOCX) [file pone.0302019.s001.docx]

**S1 Table: Diagnostic, Procedural, Medication, and Laboratory Codes Used to Identify Clinical Characteristics of Subjects with Preeclampsia**

| **Diagnostic Codes** | |
| --- | --- |
| Preeclampsia | "642.73" (ICD-9-CM: "Pre-eclampsia or eclampsia superimposed on pre-existing hypertension, antepartum condition or complication"); "642.71" (ICD-9-CM: "Pre-eclampsia or eclampsia superimposed on pre-existing hypertension, delivered, with or without mention of antepartum condition"); "642.51" (ICD-9-CM: "Severe pre-eclampsia, delivered, with or without mention of antepartum condition"); "642.23" (ICD-9-CM: "Other pre-existing hypertension, complicating pregnancy, childbirth, and the puerperium, antepartum condition or complication"); "642.53" (ICD-9-CM: "Severe pre-eclampsia, antepartum condition or complication"); "642.41" (ICD-9-CM: "Mild or unspecified pre-eclampsia, delivered, with or without mention of antepartum condition"); "642.4" (ICD-9-CM: "Mild or unspecified pre-eclampsia, unspecified as to episode of care or not applicable"); "642.43" (ICD-9-CM: "Mild or unspecified pre-eclampsia, antepartum condition or complication"); "642.42" (ICD-9-CM: "Mild or unspecified pre-eclampsia, delivered, with mention of postpartum complication"); "642.54" (ICD-9-CM: "Severe pre-eclampsia, postpartum condition or complication"); "642.52" (ICD-9-CM: "Severe pre-eclampsia, delivered, with mention of postpartum complication"); "642.44" (ICD-9-CM: "Mild or unspecified pre-eclampsia, postpartum condition or complication"); "642.21" (ICD-9-CM: "Other pre-existing hypertension, complicating pregnancy, childbirth, and the puerperium, delivered, with or without mention of antepartum condition"); "O14.15" (ICD-10-CM: "Severe pre-eclampsia, complicating the puerperium"); "O14.14" (ICD-10-CM: "Severe pre-eclampsia complicating childbirth"); "O14.93" (ICD-10-CM: "Unspecified pre-eclampsia, third trimester"); "O11.4" (ICD-10-CM: "Pre-existing hypertension with pre-eclampsia, complicating childbirth"); "O14.04" (ICD-10-CM: "Mild to moderate pre-eclampsia, complicating childbirth"); "O14.94" (ICD-10-CM: "Unspecified pre-eclampsia, complicating childbirth"); "O14.13" (ICD-10-CM: "Severe pre-eclampsia, third trimester"); "O11.2" (ICD-10-CM: "Pre-existing hypertension with pre-eclampsia, second trimester"); "O14.95" (ICD-10-CM: "Unspecified pre-eclampsia, complicating the puerperium"); "O14.05" (ICD-10-CM: "Mild to moderate pre-eclampsia, complicating the puerperium"); "O14.03" (ICD-10-CM: "Mild to moderate pre-eclampsia, third trimester"); "O14.12" (ICD-10-CM: "Severe pre-eclampsia, second trimester"); "O11.5" (ICD-10-CM: "Pre-existing hypertension with pre-eclampsia, complicating the puerperium"); "O11.3" (ICD-10-CM: "Pre-existing hypertension with pre-eclampsia, third trimester"); "O14.90" (ICD-10-CM: "Unspecified pre-eclampsia, unspecified trimester"); "O14.02" (ICD-10-CM: "Mild to moderate pre-eclampsia, second trimester"); "O14.1" (ICD-10-CM: "Severe pre-eclampsia"); "O14.9" (ICD-10-CM: "Unspecified pre-eclampsia"); "O14.0" (ICD-10-CM: "Mild to moderate pre-eclampsia"); "O11" (ICD-10-CM: "Pre-existing hypertension with pre-eclampsia"); "O14.00" (ICD-10-CM: "Mild to moderate pre-eclampsia, unspecified trimester"); "O14.92" (ICD-10-CM: "Unspecified pre-eclampsia, second trimester"); "O11.1" (ICD-10-CM: "Pre-existing hypertension with pre-eclampsia, first trimester"); "O14.10" (ICD-10-CM: "Severe pre-eclampsia, unspecified trimester"); "O11.9" (ICD-10-CM: "Pre-existing hypertension with pre-eclampsia, unspecified trimester") |
| **Procedure Codes** | |
| Cesarean Section | “1961” (CPT: “Anesthesia for cesarean delivery only”); “1968” (CPT: “Anesthesia for cesarean delivery following neuraxial labor analgesia/anesthesia (List separately in addition to code for primary procedure performed)”); “59510” (CPT: “Routine obstetric care including antepartum care, cesarean delivery, and postpartum care”); “59514” (CPT: “Cesarean delivery only”); “59515” (CPT: “Cesarean delivery only; including postpartum care”); “59622” (CPT: “Cesarean delivery only, following attempted vaginal delivery after previous cesarean delivery; including postpartum care”); “59620” (CPT: “Cesarean delivery only, following attempted vaginal delivery after previous cesarean delivery”); “74” (ICD-9-CM: “Classical cesarean section”); “74.1” (ICD-9-CM: “Low cervical cesarean section”); “236985002” (SNOMED: “Emergency lower segment cesarean section”); “274130007” (SNOMED: “Emergency cesarean section”); |
| Critical Care Services | “99291” (CPT: “Critical care, evaluation and management of the critically ill or critically injured patient; first 30-74 minutes”); “99292” (CPT: “Critical care, evaluation and management of the critically ill or critically injured patient; each additional 30 minutes (List separately in addition to code for primary service)”); |
| Mechanical Ventilation | “94003” (CPT: “Ventilation assist and management, initiation of pressure or volume preset ventilators for assisted or controlled breathing; hospital inpatient/observation, each subsequent day”); “94002” (CPT: “Ventilation assist and management, initiation of pressure or volume preset ventilators for assisted or controlled breathing; hospital inpatient/observation, initial day”); “5A1935Z” (ICD-10-PCS: “Respiratory Ventilation, Less than 24 Consecutive Hours”); “5A1945Z” (ICD-10-PCS: “Respiratory Ventilation, 24-96 Consecutive Hours”); “0BH17EZ” (ICD-10-PCS: “Insertion of Endotracheal Airway into Trachea, Via Natural or Artificial Opening”); “5A1955Z” (ICD-10-PCS: “Respiratory Ventilation, Greater than 96 Consecutive Hours”) |
| **Medication Codes** | |
| Anti-Hypertensives | “6185” (RxNorm: “labetalol”); “5470” (RxNorm: “hydralazine”); “7417” (RxNorm: “nifedipine”); “7396” (RxNorm: “nicardipine”); “4917” (RxNorm: “nitroglycerin”); “49737” (RxNorm: “esmolol”); “7476” (RxNorm: “nitroprusside”); “409233934” (NDC: “labetalol”); “51079092920” (NDC: “labetalol”); “63323061401” (NDC: “hydralazine”); “68084059711” (NDC: “nifedipine”); “409226720” (NDC: “labetalol”); “228249710” (NDC: “nifedipine”); “68084059701” (NDC: “nifedipine”); “409226725” (NDC: “labetalol”); “904592961” (NDC: “labetalol”); “63739036610” (NDC: “labetalol”); “60687011401” (NDC: “labetalol”); “43386044024” (NDC: “nifedipine”); “68682010910” (NDC: “nifedipine”); “904592861” (NDC: “labetalol”); “51079092820” (NDC: “labetalol”); “185011701” (NDC: “labetalol”); “69265066” (NDC: “nifedipine”); “143968910” (NDC: “nicardipine”); “50268059915” (NDC: “nifedipine”); “904644161” (NDC: “hydralazine”); “51079007520” (NDC: “hydralazine”); “59762669103” (NDC: “nifedipine”); “185001001” (NDC: “labetalol”); “63323061455” (NDC: “hydralazine”); “143962301” (NDC: “labetalol”); “409226754” (NDC: “labetalol”); “71041813” (NDC: “nitroglycerin”); “68084059801” (NDC: “nifedipine”); “68682010510” (NDC: “nifedipine”); “50742062101” (NDC: “nifedipine”); “68084060321” (NDC: “nifedipine”); “24979001101” (NDC: “nifedipine”); “47781058629” (NDC: “labetalol”); “904644061” (NDC: “hydralazine”); “185011805” (NDC: “labetalol”); “58657060501” (NDC: “labetalol”); “58657060601” (NDC: “labetalol”); “378048001” (NDC: “nifedipine”); “378048101” (NDC: “nifedipine”); “69260066” (NDC: “nifedipine”); “69266066” (NDC: “nifedipine”); “62584073301” (NDC: “hydralazine”); “68084002201” (NDC: “nifedipine”); “185011801” (NDC: “labetalol”); “68001020500” (NDC: “labetalol”); “68084044701” (NDC: “hydralazine”); “591060501” (NDC: “labetalol”); “50742026101” (NDC: “nifedipine”); “143962201” (NDC: “labetalol”); “281032608” (NDC: “nitroglycerin”); “207773” (RxNorm: “nifedipine”); “896762” (RxNorm: “labetalol”); “207772” (RxNorm: “nifedipine”); “896758” (RxNorm: “labetalol”); “905222” (RxNorm: “hydralazine”); “207774” (RxNorm: “nifedipine”); “198035” (RxNorm: “nifedipine”); “896766” (RxNorm: “labetalol”); “896771” (RxNorm: “labetalol”); “966571” (RxNorm: “hydralazine”); “1234256” (RxNorm: “labetalol”); “896781” (RxNorm: “labetalol”); “63323061416” (NDC: “hydralazine”); “69315021101” (NDC: “nifedipine”); “76282031010” (NDC: “hydralazine”); “69265041” (NDC: “nifedipine”); “69267066” (NDC: “nifedipine”); “69266041” (NDC: “nifedipine”); “68682010530” (NDC: “nifedipine”); “68682010630” (NDC: “nifedipine”); “71930003552” (NDC: “labetalol”); “378049401” (NDC: “nifedipine”); “68001020403” (NDC: “labetalol”); “68682010710” (NDC: “nifedipine”); “62175026037” (NDC: “nifedipine”); “67457018210” (NDC: “esmolol”); “198034” (RxNorm: “nifedipine”); “858603” (RxNorm: “nicardipine”); “858613” (RxNorm: “nicardipine”); “62175026237” (NDC: “nifedipine”); “68682010610” (NDC: “nifedipine”); “10019005561” (NDC: “esmolol”); “69292050310” (NDC: “labetalol”); “378035301” (NDC: “nifedipine”); “378036001” (NDC: “nifedipine”); “50742026201” (NDC: “nifedipine”); “24979001001” (NDC: “nifedipine”); “50742062201” (NDC: “nifedipine”); “71930003652” (NDC: “labetalol”); “69265072” (NDC: “nifedipine”); “68682010830” (NDC: “nifedipine”); “62584073401” (NDC: “hydralazine”); “198036” (RxNorm: “nifedipine”); “198032” (RxNorm: “nifedipine”); “62175026246” (NDC: “nifedipine”); “338104902” (NDC: “nitroglycerin”); “50742062001” (NDC: “nifedipine”); “69292050110” (NDC: “labetalol”); “69266072” (NDC: “nifedipine”); “228253010” (NDC: “nifedipine”); “68682010810” (NDC: “nifedipine”); “10122032510” (NDC: “nicardipine”); “25021031002” (NDC: “nitroprusside”); “49884012401” (NDC: “labetalol”); “62175026137” (NDC: “nifedipine”); “69315021201” (NDC: “nifedipine”); “905395” (RxNorm: “hydralazine”); “1812011” (RxNorm: “nifedipine”); “905199” (RxNorm: “hydralazine”); “905225” (RxNorm: “hydralazine”); “1812013” (RxNorm: “nifedipine”); “858601” (RxNorm: “nicardipine”); “1812015” (RxNorm: “nifedipine”); “312004” (RxNorm: “nitroglycerin”); “858605” (RxNorm: “nicardipine”); “858607” (RxNorm: “nicardipine”); “242946” (RxNorm: “nitroglycerin”); “198033” (RxNorm: “nifedipine”) |
| Aspirin | “1191” (RxNorm: “aspirin”); “904404073” (NDC: “aspirin”); “63739043401” (NDC: “aspirin”); “536100836” (NDC: “aspirin”); “63739052201” (NDC: “aspirin”); “63739052301” (NDC: “aspirin”); “363058714” (NDC: “aspirin”); “62107002726” (NDC: “aspirin”); “113027468” (NDC: “aspirin”); “904671318” (NDC: “aspirin”); “536100410” (NDC: “aspirin”); “243670” (RxNorm: “aspirin”); “318272” (RxNorm: “aspirin”); “308416” (RxNorm: “aspirin”); “363056314” (NDC: “aspirin”); “62107002732” (NDC: “aspirin”); “70000017001” (NDC: “aspirin”); “904679480” (NDC: “aspirin”); “198467” (RxNorm: “aspirin”); “212033” (RxNorm: “aspirin”) |
| Corticosteroids | “1514” (RxNorm: “betamethasone”); “3264” (RxNorm: “dexamethasone”); “63323016501” (NDC: “dexamethasone”); “63323050601” (NDC: “dexamethasone”); “517072001” (NDC: “betamethasone”); “641036725” (NDC: “dexamethasone”); “63323051610” (NDC: “dexamethasone”); “168005515” (NDC: “betamethasone”); “65853302” (NDC: “dexamethasone”); “51672127401” (NDC: “betamethasone”); “309696” (RxNorm: “dexamethasone”); “578803” (RxNorm: “betamethasone”); “578806” (RxNorm: “betamethasone”); “63323016505” (NDC: “dexamethasone”); “197580” (RxNorm: “dexamethasone”); “197582” (RxNorm: “dexamethasone”); “205712” (RxNorm: “dexamethasone”); “403908” (RxNorm: “dexamethasone”); “1812194” (RxNorm: “dexamethasone”); “226343” (RxNorm: “dexamethasone”); “1116927” (RxNorm: “dexamethasone”) |
| Magnesium Sulfate | “6585” (RxNorm: “magnesium sulfate”); “409672923” (NDC: “magnesium sulfate”); “63323010601” (NDC: “magnesium sulfate”); “409672903” (NDC: “magnesium sulfate”); “409672924” (NDC: “magnesium sulfate”); “63323006402” (NDC: “magnesium sulfate”); “409672723” (NDC: “magnesium sulfate”); “409673013” (NDC: “magnesium sulfate”); “63323010615” (NDC: “magnesium sulfate”); “63323010705” (NDC: “magnesium sulfate”); “63323010605” (NDC: “magnesium sulfate”); “409175410” (NDC: “magnesium sulfate”); “63323010801” (NDC: “magnesium sulfate”); “63323064220” (NDC: “magnesium sulfate”); “63323006410” (NDC: “magnesium sulfate”); “51754100004” (NDC: “magnesium sulfate”); “1658262” (RxNorm: “magnesium sulfate”); “1658264” (RxNorm: “magnesium sulfate”); “1658259” (RxNorm: “magnesium sulfate”); “829757” (RxNorm: “magnesium sulfate”); “1658265” (RxNorm: “magnesium sulfate”); “829734” (RxNorm: “magnesium sulfate”); “829762” (RxNorm: “magnesium sulfate”); “1658236” (RxNorm: “magnesium sulfate”) |
| **Laboratory Codes** | |
| Alanine aminotransferase [Enzymatic activity/volume] in Serum, Plasma or Blood | “1743-4” (LOINC: “Alanine aminotransferase [Enzymatic activity/volume] in Serum or Plasma by With P-5'-P”); “77144-4” (LOINC: “Alanine aminotransferase [Enzymatic activity/volume] in Serum, Plasma or Blood”); “1744-2” (LOINC: “Alanine aminotransferase [Enzymatic activity/volume] in Serum or Plasma by No addition of P-5'-P”); “1742-6” (LOINC: “Alanine aminotransferase [Enzymatic activity/volume] in Serum or Plasma”); “76625-3” (LOINC: “Alanine aminotransferase [Enzymatic activity/volume] in Blood”) |
| Aspartate aminotransferase [Enzymatic activity/volume] in Serum or Plasma | “30239-8” (LOINC: “Aspartate aminotransferase [Enzymatic activity/volume] in Serum or Plasma by With P-5'-P”); “1920-8” (LOINC: “Aspartate aminotransferase [Enzymatic activity/volume] in Serum or Plasma”) |
| Creatinine [Mass/volume] in Serum, Plasma or Blood | “38483-4” (LOINC: “Creatinine [Mass/volume] in Blood”); “2160-0” (LOINC: “Creatinine [Mass/volume] in Serum or Plasma”) |
| Hematocrit [Volume Fraction] of Blood | “4544-3” (LOINC: “Hematocrit [Volume Fraction] of Blood by Automated count”); “31100-1” (LOINC: “Hematocrit [Volume Fraction] of Blood by Impedance”); “20570-8” (LOINC: “Hematocrit [Volume Fraction] of Blood”); “4545-0” (LOINC: “Hematocrit [Volume Fraction] of Blood by Centrifugation”); “48703-3” (LOINC: “Hematocrit [Volume Fraction] of Blood by Estimated”) |
| Platelets [#/volume] in Blood | “26515-7” (LOINC: “Platelets [#/volume] in Blood”); “777-3” (LOINC: “Platelets [#/volume] in Blood by Automated count”); “778-1” (LOINC: “Platelets [#/volume] in Blood by Manual count”); “49497-1” (LOINC: “Platelets [#/volume] in Blood by Estimate”) |
| Urate [Mass/volume] in Serum or Plasma | "3084-1 " (LOINC: "Urate [Mass/volume] in Serum or Plasma") |
| **Vital Sign Codes** | |
| Blood Pressure Systolic | “75997-7” (LOINC: “Systolic blood pressure by Continuous non-invasive monitoring”); “76215-3” (LOINC: “Invasive Systolic blood pressure”); “76534-7” (LOINC: “Systolic blood pressure by Noninvasive”); “8450-9” (LOINC: “Systolic blood pressure--expiration”); “8451-7” (LOINC: “Systolic blood pressure--inspiration”); “8452-5” (LOINC: “Systolic blood pressure.inspiration - expiration”); “8459-0” (LOINC: “Systolic blood pressure--sitting”); “8460-8” (LOINC: “Systolic blood pressure--standing”); “8461-6” (LOINC: “Systolic blood pressure--supine”); “8479-8” (LOINC: “Systolic blood pressure by palpation”); “8480-6” (LOINC: “Blood Pressure Systolic”); “87739-9” (LOINC: “Systolic blood pressure--W exercise”); “87741-5” (LOINC: “Systolic blood pressure--post exercise”) |
| Body Mass Index | LOINC: “39156-5” (LOINC: “Body Mass Index”) |

ICD-9-CM (International Classification of Diseases, 9th Edition); ICD-10-CM (International Classification of Diseases, 10th Edition); LOINC Logical Observation Identifiers Names and Codes (LOINC); CPT, Common Procedural Terminology; NDC, National Drug Code
