# Supplementary material for: Impact of hyponatremia in preeclamptic patients with severe features
Source: PLoS One. 2024 Jul 8;19(7):e0302019. doi: 10.1371/journal.pone.0302019 (PMC11230559; doi:10.1371/journal.pone.0302019)
Supplement: S2 Table — (DOCX) [file pone.0302019.s002.docx]

**S2 Table: Interventions Applied In Subjects With Preeclampsia Divided By Year of Preeclampsia Diagnosis**

|  | **Na Level of 134 mEq/L and Below**  **(n = 215)** | | | | | | | **Na Level of 135 mEq/L and Above**  **(n = 2686)** | | | | | | |
| --- | --- | --- | --- | --- | --- | --- | --- | --- | --- | --- | --- | --- | --- | --- |
| Year of Diagnosis | 2015  (n = 2) | 2016  (n = 11) | 2017  (n = 28) | 2018  (n = 50) | 2019  (n = 75) | 2020  (n = 47) | 2021  (n = 2) | 2015  (n = 21) | 2016  (n = 310) | 2017  (n = 497) | 2018  (n = 640) | 2019  (n = 759) | 2020  (n = 437) | 2021  (n = 22) |
| Emergency  Anti-Hypertensives | 2 (100%) | 8 (72.7%) | 17 (60.7%) | 35 (70.0%) | 61 (81.3%) | 40 (85.1%) | 2 (100%) | 19 (90.5%) | 184 (59.4%) | 319 (64.2%) | 324 (50.6%) | 566 (74.6%) | 381 (87.2%) | 18 (81.8%) |
| Aspirin | 0 (0%) | 1 (9.1%) | 1 (3.6%) | 3 (6.0%) | 5 (6.7%) | 2 (4.3%) | 0 (0%) | 0 (0%) | 21 (6.8%) | 22 (4.4%) | 17 (2.7%) | 47 (6.2%) | 31 (7.1%) | 1 (4.5%) |
| Antenatal Steroids | 2 (100%) | 5 (45.5%) | 9 (32.1%) | 28 (56.0%) | 35 (46.7%) | 23 (48.9%) | 1 (50.0%) | 4 (19.0%) | 120 (38.7%) | 153 (30.8%) | 171 (26.7%) | 321 (42.3%) | 172 (39.4%) | 12 (54.5%) |
